# Mild knee osteoarthritis predicts dissatisfaction after total knee arthroplasty: a prospective study of 186 patients aged 65 years or less with 2-year follow-up

**DOI:** 10.1186/s12891-021-04543-8

**Published:** 2021-08-05

**Authors:** Sanni Leppänen, Mika Niemeläinen, Heini Huhtala, Antti Eskelinen

**Affiliations:** 1 Coxa Hospital for Joint Replacement, Tampere, Finland; 2 Faculty of Medicine and Health Technologies, Tampere University, Tampere, Finland; 3 Faculty of Social Sciences, Tampere University, Tampere, Finland

**Keywords:** Knee arthroplasty, Outcome, Satisfaction, Pain, 65 years or less, Young patients, Patient-reported outcome measures

## Abstract

**Background and aims:**

The incidence of total knee arthroplasty (TKA) is increasing, especially among younger working-age patients. However, dissatisfaction rates in this population are higher than among older patients. The aim of this study was to assess the rates of dissatisfaction and persistent pain after TKA and to evaluate those factors that predict these outcomes.

**Material and methods:**

In total, 186 patients undergoing unilateral TKA aged 65 years or less were enrolled into this prospective observational study with 2-year follow-up. To assess the outcome, the visual analogue scales regarding satisfaction and persistent pain at rest and during exercise were used. In addition, the association between patients´ demographics, radiographic severity of knee osteoarthritis (OA), patient-reported outcome measures (PROMs) and dissatisfaction and persistent pain were tested by univariate logistic regression analysis. Mild OA was defined as Kellgren-Lawrence (KL) grade 2 and severe OA as KL grade 3–4. Furthermore, multiple logistic regression analysis was also conducted to test statistically significant relations.

**Results:**

After 2 years, 12 % (*n* = 23) of patients were dissatisfied with the outcome of TKA, 27 % (*n* = 50) reported persistent pain during exercise and 10 % (*n* = 18) at rest. Patients with mild knee OA were significantly more dissatisfied (28.6 %) than patients with more severe OA (8.7 %) (*p* = 0.003). Younger patients had an increased risk for both dissatisfaction and persistent pain. Apart from KOOS Quality of Life, poor preoperative KOOS subscores were also predictive for these outcomes.

**Conclusion:**

Mild radiographic knee OA was the main predicting factor for dissatisfaction after TKA. Thus, performing TKA for such patients should be carefully considered. Furthermore, these patients should be informed about the increased risk for dissatisfaction and the same seems to apply to younger patients. Interestingly, when TKA is performed for patients with more severe knee OA, the satisfaction rates seem to be somewhat higher than those previously reported.

**Trial registration:**

The study was retrospectively registered with ClinicalTrials.gov (registration number NCT03233620) on 28 July 2017.

## Background

Recent studies clearly indicate that the incidence of total knee arthroplasty (TKA) will increase in the near future [1–3]. Indeed, an increasing number of TKAs are being performed not only for older patients but also for younger patients, and the largest proportional increase is reported to be in patients younger than 65 years of age [2]. While TKA has been shown to be an effective treatment for end-stage knee osteoarthritis (OA), younger age is known to be associated with an increased risk for both adverse outcomes and revision surgery [4–6]. Furthermore, younger patients often have high expectations for the outcome of TKA, and this may predispose them to dissatisfaction after the operation [7–10]. The aim of this prospective observational study was to assess which preoperative factors predict dissatisfaction in patients aged 65 years or less undergoing TKA. Primary outcomes were satisfaction and persistent pain as measured with the visual analogue scale (VAS).

## Materials and methods

For the original 2-year prospective cohort study assessing the results of unilateral and bilateral TKAs and UKAs, 255 patients scheduled for knee arthroplasty were enrolled between 1st March 2012 and 30th October 2014 at our high-volume academic tertiary referral centre [11].

The inclusion criteria of the original study cohort were as follows: (1) Age 65 or less and (2) scheduled for knee arthroplasty for primary OA [11]. The exclusion criteria were as follows: (1) rheumatoid arthritis or other inflammatory diseases (2) post-traumatic OA (3) unwilling to provide informed consent (4) physical, mental, or neurological conditions that could compromise the patient´s ability and compliance with postoperative rehabilitation and follow-up (e.g., drug or alcohol abuse, serious mental illness, general neurological conditions, such as Parkinson’s disease and multiple sclerosis) (5) known sensitivity to the materials used in the devices [11].

### Patient flow

**Fig. 1:**
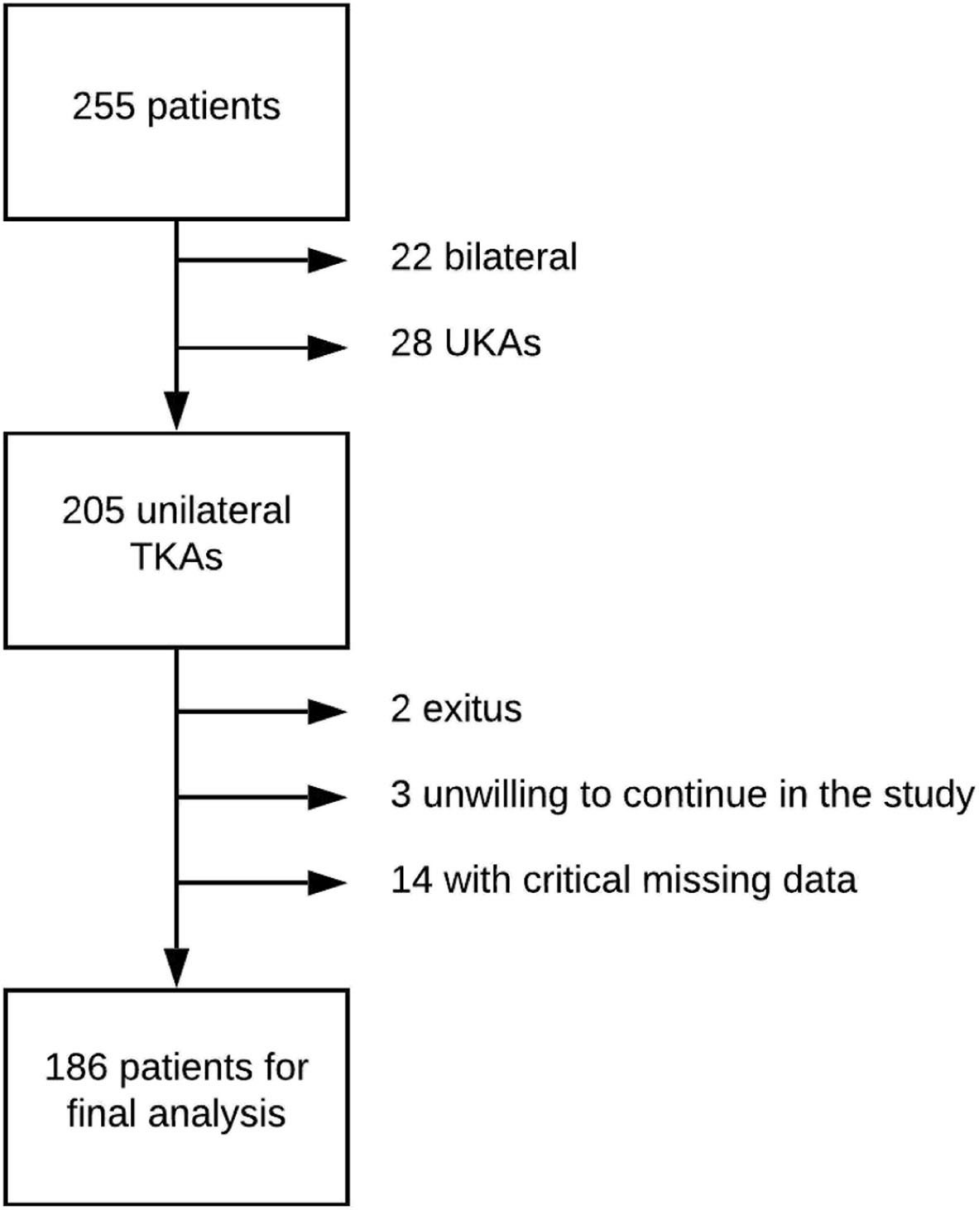
Patient flow diagram

For the purposes of this study, bilateral TKAs and unicompartmental knee arthroplasties were excluded, leaving 205 unilateral TKA patients to be included. Two patients (1 %) died during follow-up. Three patients did not return questionnaires, despite repeated requests, and were therefore considered unwilling to continue in the study. Thus, two hundred patients were available for the 2-year follow up visits, which were carried out via questionnaires by mail. However, 14 patients (7 %) returned only partially completed questionnaires and had left the visual analogue scales regarding satisfaction and persistent pain. These patients were also excluded, and the analysis of the final results was based on 186 (91 %) TKAs (Fig. 1).

All operations were performed by senior orthopaedic surgeons, and all patients were treated with the same routine postoperative rehabilitation and pain management protocol.

### Implants used

The 186 patients underwent cemented TKA using either PFC (151 knees; DePuy Synthes, Warsaw, IN) or Nexgen (35 knees; Zimmer Biomet, Warsaw, IN). In 8 (4 %) of the TKAs, the patella was resurfaced due to perioperative maltracking. Primary implant was cruciate retaining (CR) model in all patients but posterior stabilised (PS) implants were used if PCL insufficiency was detected peroperatively. Of the 186 TKAs, 177 (95 %) were CR and the remaining 9 were PS.

### Outcome measures

The visual analogue scale (VAS) was measured with a scale from 0 to 100. It is widely used for evaluating pain intensity and in this study was used to evaluate pain both at rest and during exercise. Satisfaction regarding the studied knee was also measured with VAS as was done by Scott et al. and it is shown to be a reliable method for measuring satisfaction [9, 12]. All VASs were collected pre- and 2 years postoperatively. When evaluating pain, a score of 100 indicated the worst possible pain. When evaluating satisfaction, a score of 100 indicated the best possible satisfaction. In univariate analysis, dissatisfied patients were defined as satisfaction VAS ≤ 50 and patients experiencing pain as pain VAS > 30, respectively [9, 13]. The minimal clinical important difference (MCID) for measuring pain with VAS is 10 points [13].

Patients were asked to fill out a background questionnaire that included information on working status and physical activities. Medical comorbidities were asked as “Do you have any other conditions that limit your physical activity more than your diagnosed knee osteoarthritis? Yes/No”. Patients were also asked to fill three additional PROMs both pre- and postoperatively: the Oxford Knee Score (OKS) [14], the Knee injury and Osteoarthritis Outcome Score (KOOS) [15] and the RAND 36-Item Health Survey (RAND-36) [16].

The severity of knee OA was assessed from preoperative standing fixed flexion view (FFV) radiographs using the Kellgren-Lawrence (KL) classification [17]. Mild knee OA was defined as KL grade 2 and severe knee OA as KL grade 3-4 [17]. The varus/valgus alignment was assessed from long-leg radiographs.

### Statistical analysis

Demographic data were presented as the median with quartiles (Q_1_ to Q_3_), range (min to max), or as mean (CI 95 %). The KOOS scores were divided into five subscale scores (pain, other symptoms, function in daily living (ADL), function in sport and knee related quality of life (QoL)) and were analysed separately. RAND-36 questionnaire scores were divided into two subscale scores (Physical Component Score (PCS) and Mental Component Score (MCS)) and were analysed separately. Univariate logistic regression was used to evaluate the association between the preoperative factors and dissatisfaction and persistent pain. Fisher’s exact test was used to compare the dissatisfaction rates, the achieved MCID rates and the prevalence of comorbidities between the KL 2 and KL 3–4 subgroups. Multiple logistic regression analysis was also used to compare the dissatisfaction rates between these two KL subgroups adjusted with age, gender and BMI, which are known or potential risk factors for more severe knee OA, and were thus considered as confounding factors [18–21]. A *p*-value less than 0.05 was considered statistically significant. Data were analysed using the SPSS (version 26) statistical package (IBM, Armonk, NY, USA.).

The study was retrospectively registered with ClinicalTrials.gov (registration number NCT03233620) on 28/07/2017. All methods were performed according to the relevant guidelines and regulations and the reporting guideline Strobe was used [22, 23].

## Results

Patients´ demographics are shown in Table 1.

**Table 1:** Patient (*n* = 186) demographics

	median/n	range/%
Age (years)	60	28–65
BMI	31	20–54
Females	117	63
Kellgren-Lawrence		
KL 2	36	19
KL 3	87	47
KL 4	63	34
Preoperative varus alignment	127	68
At work preoperatively	92	50
Comorbidities limiting physical activity	28	15

### Dissatisfaction: prevalence and relations to demographics

At the time of the 2-year follow-up, 12.4 % (*n* = 23) of the patients were dissatisfied with their operated knee. Those patients with mild radiographic knee OA were significantly more dissatisfied than patients with more severe knee OA (28.6 % vs. 8.7 %, *p* = 0.003; Table 2), and the risk for dissatisfaction among these patients was more than 4.2-fold (OR 4.22, 95 % CI 1.67–10.66, *p* = 0.002; Table 3). Furthermore, this difference also persisted in the multiple logistic regression analysis adjusted with age, gender and BMI (OR 4.58, 95 % CI 1.72–12.20, *p* = 0.002). There was also no difference in prevalence of comorbidities limiting physical activity between mild and severe OA subgroups (17,1 % vs. 15.0 %, *p* = 0.795). Patients with more severe knee OA also showed a trend to more often achieve clinically significant improvement (exceeding MCID) in VAS satisfaction between the preoperative and the 2-year measurements. This finding, however, lacked statistical significance (Table 2). Younger age also significantly increased the risk for dissatisfaction (OR 0.92, 95 % CI 0.85–0.99, *p* = 0.029).

**Table 2:** Proportion of dissatisfied patients and MCID (∆10 points) in different KL-groups measured with satisfaction VAS (0-100)

	Kellgren-Lawrence (KL) classification				*p*-value
	2		3-4		
	*n*=35		*n*=150		
	%	n	%	n	
Dissatisfied	28.6	10	8.7	13	0.003
MCID^a^	90.0	27	97.2	140	0.100

^a^Proportion of patients whose satisfaction with their knee improved more than MCID (10 points in VAS satisfaction) from the preoperative evaluation to the 2-year follow-up

**Table 3:** Binary logistic regression analysis to assess the association between preoperative factors and dissatisfaction and persistent pain after TKA

Variable	Dissatisfaction			Persistent pain during exercise			Persistent pain at rest		
	OR	95% CI	*p*	OR	95% CI	*p*	OR	95% CI	*p*
Gender									
Female	1			1			1		
Male	1.64	0.68 – 3.96	0.268	0.80	0.41 – 1.56	0.526	1.06	0.39 – 2.89	0.903
Higher BMI	0.98	0.90 – 1.06	0.588	1.02	0.96 – 1.08	0.569	1.03	0.95 – 1.12	0.513
Older age	*0.92*	*0.85 – 0.99*	*0.029*	*0.93*	*0.87 – 0.99*	*0.022*	*0.84*	*0.77 – 0.93*	*<0.001*
At work	0.53	0.21 – 1.33	0.178	0.80	0.42 – 1.54	0.507	1.77	0.43 - 7.34	0.434
Alignment									
Varus	1			1			1		
Valgus	0.86	0.23 – 3.19	0.818	1.01	0.41 – 2.50	0.976	1.95	0.56 – 6.82	0.296
KL-grade									
3-4	1			1			1		
2	*4.22*	*1.67 – 10.66*	*0.002*	1.51	0.69 – 3.33	0.306	1.85	0.61 – 5.62	0.275
Comorbidities									
No	1			1			1		
Yes	1.63	0.55 – 4.83	0.377	0.92	0.36 – 2.33	0.855	0.68	0.15 – 3.12	0.615

### Persistent pain: prevalence and relations to demographics

At 2 years postoperatively, 27 % (*n* = 50) of patients reported persistent knee pain during exercise and 10 % (*n* = 18) at rest, respectively. Younger age was significantly associated with persistent pain both during exercise (OR 0.93, 95 % CI 0.87–0.99, *p* = 0.022) and at rest (OR 0.84, 95 % CI 0.77–0.93, *p* < 0.001). Other demographic factors had no effect (Table 3).

### PROMs relations to dissatisfaction and persistent pain

Using univariate logistic regression analysis, we also assessed whether preoperative PROMs predicted dissatisfaction and persistent pain at 2 years postoperatively (Table 4). We found that the more pain the patient experienced and the more symptomatic the knee was preoperatively (according to KOOS pain and symptoms subscores), the less likely they were to be satisfied and the more likely they were to suffer from persistent pain at 2 years. Also, weaker sports function (KOOS sport) and function in daily living (KOOS ADL) were statistically significantly related to both dissatisfaction and persistent pain. We also found lower OKS to be associated with persistent pain at rest (OR 0.88, CI 95 % 0.81–0.96, *p* = 0.004) and similar tendencies were also found regarding dissatisfaction and persistent pain during exercise. Both poor mental and physical state, measured with RAND-36 MCS and PCS, were associated with persistent pain at rest, the latter also exceeding the threshold for statistical difference. In addition, lower RAND-36 PCS showed a similar tendency with dissatisfaction and persistent pain during exercise. As we found age to be associated with dissatisfaction and persistent pain, we conducted also age adjusted multiple logistic regression analysis to further assess the relations between preoperative PROMs and dissatisfaction and persistent pain. All the reported findings persisted, apart from the relations between RAND-36 and persistent pain at rest, as the statistical significance was lost (OR 0.97, CI 95 % 0.93–1.00, *p* = 0.076).

**Table 4:** Association of preoperative PROMs with dissatisfaction and persistent pain after TKA

Variable	Dissatisfaction			Persistent pain during exercise			Persistent pain at rest		
	OR	95% CI	*p*-value	OR	95% CI	*p*-value	OR	95% CI	*p*-value
OKS	0.94	0.87 – 1.01	0.067	0.95	0.90 – 1.00	0.062	*0.88*	*0.81 – 0.96*	*0.004*
KOOS pain	*0.96*	*0.93 – 0.99*	*0.003*	*0.97*	*0.95 – 0.99*	*0.004*	*0.95*	*0.91 – 0.98*	*0.002*
KOOS symptoms	*0.96*	*0.93 – 0.99*	*0.003*	*0.97*	*0.94 – 0.99*	*0.002*	*0.95*	*0.91 – 0.98*	*0.002*
KOOS ADL	*0.97*	*0.94 – 0.99*	*0.011*	*0.98*	*0.96 – 1.00*	*0.045*	*0.97*	*0.94 – 0.99*	*0.022*
KOOS sport	*0.95*	*0.91 – 0.99*	*0.032*	*0.95*	*0.92 – 0.98*	*0.002*	*0.94*	*0.89 – 0.99*	*0.040*
KOOS QoL	0.99	0.96 – 1.02	0.629	0.98	0.96 – 1.00	0.108	0.98	0.95 – 1.02	0.358
RAND-36 MCS	0.99	0.97 – 1.01	0.247	0.99	0.98 – 1.01	0.208	0.98	0.96 – 1.00	0.054
RAND-36 PCS	0.97	0.94 – 1.00	0.080	0.98	0.96 – 1.00	0.065	*0.96*	*0.93 – 1.00*	*0.047*

## Discussion

The number of TKAs is rapidly increasing, especially among younger patients [24]. This may be due to the decreasing numbers of high tibial osteotomy and arthroscopic surgery of the degenerative knee [25, 26]. Thus, patients with milder knee OA may nowadays be more often referred to knee arthroplasty. Previous studies have persistently shown that a varying percentage of patients undergoing TKA end up being unsatisfied with their operated knee [9, 27, 28]. In a systematic review of TKA in patients younger than 55 years of age the overall satisfaction rate was 86 % [29]. In our study, 87 % of patients who responded were satisfied with their operated knee 2 years postoperatively, which is similar also to the satisfaction rates reported by Parvizi et al. (90 %), but somewhat higher compared to the satisfaction rates reported by Klit et al. (71 %) [10, 27]. Niemeläinen et al. reported a satisfaction rate of 85 % two years after surgery among patients aged less than 65 years comprising not only the patients of this study but also patients with unicompartmental and bilateral knee arthroplasties [11].

### Dissatisfaction and radiographic OA

We found that dissatisfaction was significantly more common among patients with milder radiographic OA – 29 % in patients with KL 2 OA and only around 9 % among patients with more severe OA. This finding is supported by Scott et al., who reported that over 50 % of patients with KL 2 OA are dissatisfied after TKA [9]. A systematic review by Nakano et al. assessing dissatisfaction after TKA in patients of all ages had a similar finding as well [30]. Also Niemeläinen et al. found mild radiographic OA to be related to dissatisfaction [11]. We also found a trend for the KL3-4 subgroup to more often achieve clinically significant improvement in satisfaction (Table 2). This finding, however, lacked statistical significance, which may be due in part to the small size of the KL 2 subgroup and thus insufficient statistical power. Patients with milder OA tend to have comorbidities, such as depression, lower back pain and fibromyalgia, more often than those who have more severe OA [31]. These conditions are all causes of psychological distress, and thus lead to difficulties in coping with pain [32]. In our study there was no difference in the prevalence of comorbidities between KL subgroups, though patients reported only conditions that limited their physical activity more than their knee OA. In previous studies, milder OA has also been associated with worse functional outcomes after TKA and an increased risk for revision surgery [33, 34]. Overall, this evidence strongly suggests that TKA should be mainly performed for patients with KL 3 or 4 knee OA. Furthermore, if patients with KL 2 knee OA are scheduled for TKA, they should be thoroughly informed about the increased likelihood of dissatisfaction after such surgery.

### Dissatisfaction: age and BMI

We also found that younger age increases the risk for dissatisfaction. This finding is in line with the findings of Lange et al., who included a matched control group of older patients aged 65–75 years in their study and found patients aged 55 years or less to be more dissatisfied with their TKA (satisfaction 86 % vs. 91 %) [35]. This finding might have to do with younger patients’ higher expectations and requirements for physical activities after surgery [7–10]. Haynes et al. reported that younger patients still do achieve a clinically significant improvement after TKA, even though they tend to have less severe radiographic OA and more severe clinical symptoms than older patients before surgery [18]. Unlike Scott et al. or Nakano et al., we found that BMI had no association with dissatisfaction [9, 30]. Therefore, although obesity is a well-known risk factor for knee OA and the risk for both prosthetic joint infection and revision surgery is markedly increased among obese patients, based on this study they seem to be as satisfied with the outcome of TKA as non-obese patients[21, 36].

### Dissatisfaction, persistent pain and PROMs

Similarly to the findings of Scott et al., we also found a trend for worse preoperative OKS being associated with dissatisfaction [9]. However, the association was more significant with OKS and persistent pain at rest. Also, apart from KOOS Quality of Life, all KOOS subscores were statistically significantly related to dissatisfaction and persistent pain. These findings support the prevalent conception that severe preoperative knee pain, along with pain elsewhere in the body, predicts persistent pain after TKA [32].

In this study, multiple standardised PROMs (OKS, KOOS, RAND-36) were collected and analysed, but all in all the associations found with dissatisfaction and persistent pain seem somewhat scattered. Some previous studies have tried to identify threshold values for preoperative OKS that indicate postoperative satisfaction but found no association between these two variables [37, 38]. In our study, KOOS was more strongly associated with dissatisfaction and persistent pain than OKS. This finding may favour KOOS as a more knee symptom-specific score in screening. KOOS includes questions that cover a wider spectrum regarding pain and functional outcome compared to OKS. KOOS also assesses the effect of the knee on quality of life. Conversely, OKS is a markedly shorter questionnaire (12 vs. 36 items), and thus it is easier for patients to fill out. Moreover, OKS was also recently chosen as the preferred condition-specific instrument for the continuous evaluation of outcomes after TKA [39]. Still, altogether these findings indicate that although PROMs are commonly used in the evaluation of outcomes after knee arthroplasty, none of them are ideal in preoperatively identifying those patients who will be dissatisfied and will have persistent pain after surgery. Therefore, they should not be the main focus in the selection of patients for TKA. Even though it is important to keep evaluating patient satisfaction and surgery outcomes, it seems that instead of using a vast battery of questionnaires, simple outcome measures, such as VAS and OKS or KOOS, would be adequate, as recently proposed by an OECD working group [39].

### Limitations and strengths

Our study was a prospective observational study that used multiple standardised PROMs. We acknowledge a few limitations in our study. First, our study lacked a control group comprising patients over the age of 65. This would have enabled us to compare the results between these two age groups. Second, an additional weakness in our study was the limited sample size, which suffered from 14 (7 %) patients missing critical data. Thus, for some comparisons, our study might have been inadequately powered to find significant differences. We also did not discuss patient expectations which might have been a confounding factor. Additionally, we analysed mainly self-reported data and outcome at 2 years and therefore were not able to evaluate immediate postoperative complications, infections, tromboembolisms or other adverse events that might have affected the results. Also, comorbidities were self-reported and limited to conditions limiting patient’s physical activity more than their knee OA. Thus, mental health issues, chronic pain and other conditions predisposing to persistent pain and dissatisfaction might not have been considered. Our study also had some obvious strengths. The study population consisted of non-selected real-world patients, and therefore, along with the study setup, was equivalent to everyday clinical practice. Additional strengths of the study were the 2-year follow-up period and the high response rate – only 4 % of patients were lost to follow-up during the study.

## Conclusions

We found that a vast majority of patients aged less than 65 years clearly benefit from TKA surgery in terms of pain relief, improved function and overall satisfaction. Dissatisfaction in this age group seems to be strongly related to mild radiographic OA. Thus, TKA should primarily be performed for patients with end-stage OA. Although severe preoperative knee pain is shown to be related to both dissatisfaction and persistent pain, based on the findings of the present study, the value of PROMs in predicting these outcomes remains uncertain. Further research with a lot larger patient cohorts on PROMs’ value in predicting satisfaction is certainly needed to provide support for the surgical decision making.

## Data Availability

The datasets used and analysed during the current study are available from the corresponding author on reasonable request.
